# Will cognitive health disparity estimates change in 2030? Investigating the impact of a Middle Eastern and North African identifier in the United States

**DOI:** 10.1002/alz70860_100839

**Published:** 2025-12-23

**Authors:** Basma Tnesh, Tiffany B Kindratt

**Affiliations:** ^1^ University of Texas at Arlington, Arlington, TX, USA

## Abstract

**Background:**

In 2024, the Office of Management and Budget (OMB) updated guidelines for measuring race/ethnicity. Statistical Policy Directive 15 requires that Middle Eastern and North African (MENA) Americans have a separate checkbox from the White race group on the 2030 US Census. It is unknown how this change will alter health estimates for other racial/ethnic groups compared to Whites. Our objectives are to 1) compare the prevalence of cognitive difficulty among diverse older adults using 2020 and 2030 US Census racial/ethnic categories and 2) determine whether the odds of cognitive difficulty differs with and without a MENA identifier.

**Method:**

We used 2018‐2022 American Community Survey (ACS) data (ages >=65 years; *n* = 3,351,611). First, we categorized race/ethnicity based on the categories collected by the 2020 US Census (White, Black, AI/AN, Asian, NH/OPI, Some Other Race, Two or More Races, Hispanic/Latino). Second, we created a separate category for older adults of MENA descent using questions on ancestry and place of birth. Bivariate statistics and multivariable logistic regression models were calculated.

**Result:**

Using 2020 categories, the odds of cognitive difficulty were higher among all racial/ethnic groups compared to White older adults. Using 2030 categories, the odds of cognitive difficulty were 1.53 times greater (95% CI=1.43‐1.62) among MENA compared to White older adults. When we compared the odds of cognitive difficulty using 2020 and 2030 US Census racial/ethnic categories, only minor changes in odds were observed (e.g., 1.38 and 1.39 among Black older adults) all 95% confidence intervals overlapped indicating no differences in cognitive health disparities reported when using the new classification.

**Conclusion:**

Our results highlight the disparity in cognitive health among MENA and White older adults. Adding a separate MENA identifier did not alter health disparities between other minority groups and Whites. Including a separate MENA checkbox on the ACS starting in 2027 is critical for moving forward discussions on health equity among older adults.

